# Formulation Development, Optimization by Box–Behnken Design, and In Vitro and Ex Vivo Characterization of Hexatriacontane-Loaded Transethosomal Gel for Antimicrobial Treatment for Skin Infections

**DOI:** 10.3390/gels9040322

**Published:** 2023-04-11

**Authors:** Alhussain H. Aodah, Sana Hashmi, Naseem Akhtar, Zabih Ullah, Ameeduzzafar Zafar, Randa Mohammed Zaki, Shamshir Khan, Mohammad Javed Ansari, Talha Jawaid, Aftab Alam, Md Sajid Ali

**Affiliations:** 1 Department of Pharmaceutics, College of Pharmacy, Prince Sattam Bin Abdulaziz University, Al Kharj 11942, Saudi Arabia; 2 Department of Pharmaceutical Sciences, Unaizah College of Pharmacy, Qassim University, Unaizah 51911, Saudi Arabia; 3 Department of Pharmaceutics, College of Dentistry and Pharmacy, Buraydah Private Colleges, Buraydah 51418, Saudi Arabia; 4 Department of Pharmaceutics, College of Pharmacy, Jouf University, Sakaka 72341, Saudi Arabia; 5 Department of Pharmaceutics and Industrial Pharmacy, Faculty of Pharmacy, Beni-Suef University, Beni-Suef 62514, Egypt; 6 Department of Pharmacognosy and Pharmaceutical Chemistry, College of Dentistry and Pharmacy, Buraydah Private Colleges, Buraydah 51418, Saudi Arabia; 7 Department of Pharmacology, College of Medicine, Imam Mohammad Ibn Saud Islamic University (IMSIU), Riyadh 13317, Saudi Arabia; 8 Department of Pharmacognosy, College of Pharmacy, Prince Sattam Bin Abdulaziz University, Al Kharj 11942, Saudi Arabia; 9 Department of Pharmaceutics, College of Pharmacy, Jazan University, Jazan 45142, Saudi Arabia

**Keywords:** transethosomes, hexatriacontane, Gram-negative pathogens (GNP), Gram-positive pathogens (GPP), antibacterial activity

## Abstract

There are many different infections and factors that can lead to skin illnesses, but bacteria and fungi are the most frequent. The goal of this study was to develop a hexatriacontane-loaded transethosome (HTC-TES) for treating skin conditions caused by microbes. The HTC-TES was developed utilizing the rotary evaporator technique, and Box–Behnken design (BBD) was utilized to improve it. The responses chosen were particle size (nm) (Y1), polydispersity index (PDI) (Y2), and entrapment efficiency (Y3), while the independent variables chosen were lipoid (mg) (A), ethanol (%) (B), and sodium cholate (mg) (C). The optimized TES formulation with code F1, which contains lipoid (mg) (A) 90, ethanol (%) (B) 25, and sodium cholate (mg) (C) 10, was chosen. Furthermore, the generated HTC-TES was used for research on confocal laser scanning microscopy (CLSM), dermatokinetics, and in vitro HTC release. The results of the study reveal that the ideal formulation of the HTC-loaded TES had the following characteristics: 183.9 nm, 0.262 mV, −26.61 mV, and 87.79% particle size, PDI, and entrapment efficiency, respectively. An in vitro study on HTC release found that the rates of HTC release for HTC-TES and conventional HTC suspension were 74.67 ± 0.22 and 38.75 ± 0.23, respectively. The release of hexatriacontane from TES fit the Higuchi model the best, and the Korsmeyer–Peppas model indicates the release of HTC followed a non-Fickian diffusion. By having a higher negative value for cohesiveness, the produced gel formulation demonstrated its stiffness, whereas good spreadability indicated better gel application to the surface. In a dermatokinetics study, it was discovered that TES gel considerably increased HTC transport in the epidermal layers (*p* < 0.05) when compared to HTC conventional formulation gel (HTC-CFG). The CLSM of rat skin treated with the rhodamine B-loaded TES formulation demonstrated a deeper penetration of 30.0 µm in comparison to the hydroalcoholic rhodamine B solution (0.15 µm). The HTC-loaded transethosome was determined to be an effective inhibitor of pathogenic bacterial growth (*S. aureus* and *E. coli*) at a concentration of 10 mg/mL. It was discovered that both pathogenic strains were susceptible to free HTC. According to the findings, HTC-TES gel can be employed to enhance therapeutic outcomes through antimicrobial activity.

## 1. Introduction

More than 25% of global deaths each year are caused by infectious diseases. Damage to economies and public health, especially in underdeveloped nations, has been substantial due to emerging infectious diseases (EIDs). It is also worth noting that the threat posed by infectious diseases has grown steadily since the 1940s. Many different microorganisms, hosts, and environmental elements have a role in facilitating the spread of infectious diseases. The vast majority of infectious-disease-causing agents are bacteria [1,2,3]. Numerous dermatological disorders have mysterious origins that have yet to be understood. Diseases such as scleroderma, eczema, acne, psoriasis, dyshidrosis, seborrheic dermatitis, and rosacea have all had an infectious theory put forward for them [4]. Is it feasible that certain bacteria or shifts in the skin’s microbial ecology set off some of these diseases or keep them going? If this is the case, the microorganisms’ harmful effect could be brought about by the discharge of toxins, the invasion of cells, the modification of host-cell regulation, the generation of allergic or inflammatory reactions, or even a shift in the composition of the microbiome itself [4].

Over a hundred plant species from a wide range of plant groups have been identified for their potential therapeutic uses [5]. Plant species such as *Melastoma malabathricum* L., *Merremia borneensis* Merr., *Pandanus amaryllifolius* Roxb, and *Senna alata* (L.) Roxbare are only some of the abundant plant species that may be found in Saudi Arabia. From these four plants, many phytochemicals, including hexatriacontane, were isolated and identified [5]. Selvan, A.T. et al. (2014) conducted a study on the plant species Pupalia Lappacea, and they discovered the presence of hexatriacontane in its aerial parts and antioxidant activity of hexatriacontane [6]. According to a report by Yassa et al. (2009), hexatriacontane is one of the active ingredients in *Rosa damascena Mill*. essential oils with stronger antioxidant activity [7]. Hexatricontane was identified as an active ingredient in a study conducted by Nayak B et al. (2018) on the antioxidant and antimicrobial screening of *Suaeda maritima* L. (Dumort). The hexatriacontane was reported to have antioxidant activity, and it was also found to have a significant impact on the resistance of the Gram-negative and Gram-positive pathogens of the plant species [8]. According to the results of a study by Miller et al. (1988), who developed liposomes that encapsulated octadecane and hexatriacontane for transport-limited microbial metabolism, they have demonstrated a considerable influence on antimicrobial activity [9]. According to a disc diffusion study, the *C. roseus* contains hexatriacontane, which possesses antimicrobial activity [10]. Gamal El-Din et al. (2022) investigated the antibacterial activity of *Jatropha* species. Along with other active components, the primary volatile component of the plant species, hexatriacontane, was also discovered [11]. Although these findings did not directly link to the antimicrobial activity of hexatriacontane, the report of Miller et al. (1988) indicated that if HTC is properly loaded on some carriers, the HTC may show antimicrobial potential [9].

Due to the hydrophobic nature of hexatriacontane and its poor solubility in water, its bioavailability and biological efficacy are questionable as a therapeutic molecule [12]. A surge in studies has been seen recently on the use of nanoencapsulation techniques as a means of increasing the bioavailability of poorly bioavailable drugs and phytocompounds. Optimizing drug efficacy is a goal in the research and development of novel pharmaceutical drugs [13]. Recent developments in nanotechnology have presented a viable method for enhancing the performance and reliability of the many current antibacterial drugs. In contrast to the free drug, the antibacterial activity and selective delivery of nano-formulated antimicrobial formulations in different vesicular systems are significantly improved [14]. One intriguing strategy for improving therapeutic efficacy while reducing adverse effects is the encapsulation of antibacterial drugs in various nano-vesicular systems. The ethosome is an example of a nano-system of this category. Ethosomes can fuse with bacterial cell membranes to increase the effectiveness of antimicrobial drugs, regardless of the solubility of the drugs [15]. The perks of topical drug-delivery systems are intriguing, but traditional topical systems have difficulty penetrating deeper layers of skin. Therefore, for an efficient treatment, it is necessary for the drugs to achieve localized activity.

In recent years, researchers have tried to developed elastic vesicles to overcome these constraints. There are two forms of elastic vesicles: the lipid- and edge-activator-containing transfersomes and the lipid- and ethanol-containing ethosomes [16,17,18,19,20,21]. The edge activator and ethanol in transethosomes (TES) allow for deeper penetration and protection against skin infections, making them ideal vehicles for targeted distribution into the skin [22]. Transethosomes, which include both ethanol and edge actuators, demonstrate the benefits of both ethosomes and deformable liposomes. As TES, they can distort their shape while passing through the layers of skin and can reach the deepest layers of the skin without rupturing them. The ethanol and sodium cholate components in TES have increased the transit of drugs confined in the stratum corneum. Moreover, transethosomes showed improved deposition and penetration capabilities [23].

The primary and major objectives of this investigation were to examine the antibacterial activity of HTC against both types of Gram -ve (GNB) and Gram +ve bacteria (GPB) and to investigate the potential of HTC for the development of TES. HTC-TES gel was optimized by adjusting the amounts of lipid, ethanol, and sodium cholate used in the formulation process. One of the design of experiments (DOE) methodology’s subsets, Box–Behnken design (BBD), aids in the development of higher-order models that require fewer runs than other factorial designs. By reducing the number of trials, BBD application thus not only saves time but also lowers costs. By combining the mathematical and statistical techniques required for the optimization modelling and analysis of experiments where the output is influenced by various factors, BBD strives to optimize the output of a system with the fewest runs. Three-factor, three-level Box–Behnken design is one of best-suited optimization techniques of response surface methodology. This method is ideal for exploring second-order polynomial models of quadratic response surfaces [24]. Using Box–Behnken design (BBD) analysis, the correlations between several factors and changes in vesicle size, polydispersity index, and percentage entrapment efficiency were investigated. The vesicle morphology, HTC release in vitro, penetration properties, and dermatokinetics of the optimized HTC-TES gel formulation were also assessed. It can be well anticipated from the research that the optimized HTC-TES gel formulation would result in the delivery of HTC in the desired amount for effective antimicrobial activity.

## 2. Results and Discussion

### 2.1. Box–Behnken Design’s Optimization of Transethosomes

**Figure 1:**
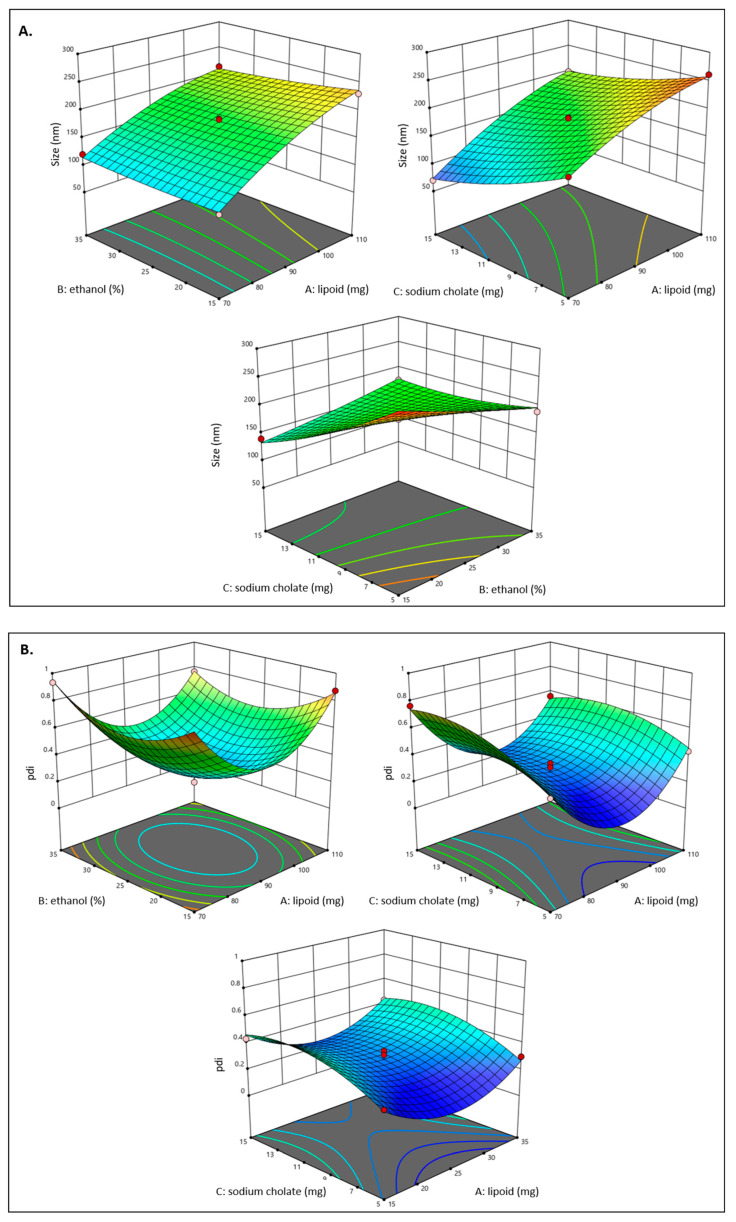

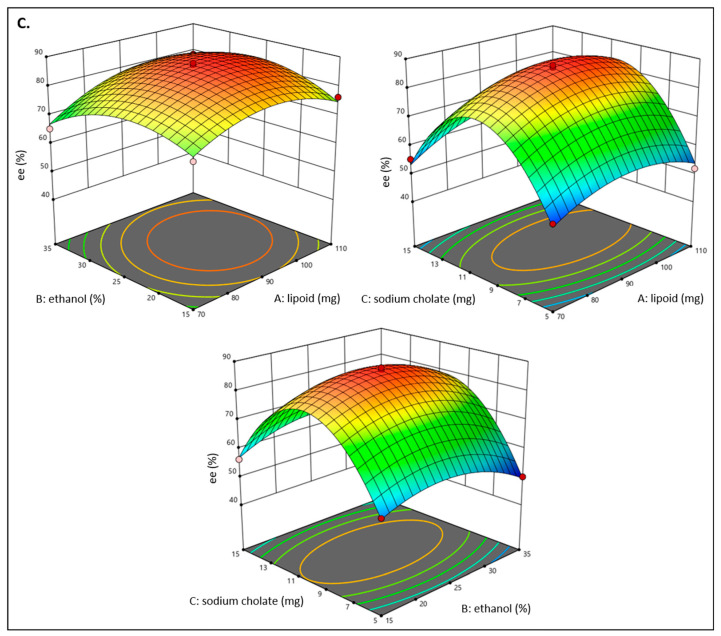
The figure shows the following: (**A**) 3D surface plot representation of the relationship between independent variables and vesicle size, (**B**) 3D surface plot representation of the relationship between independent variables and PDI, and (**C**) 3D surface plot representation of the relationship between independent variables and entrapment efficiency %.

In order to create three different formulas, the Box–Behnken design produced a total of seventeen experimental runs. In Table 1A, we can see the results of these tests. Vesicle size (Y1), polydispersity index (PDI) (Y2), and entrapment efficiency (Y3) had values in the ranges of 70.11 nm to 270.87 nm, 0.262 ± 1.12 to 0.934 ± 0.29, and 39.23 ± 0.68% to 80.14 ± 0.53% respectively. Results from all 17 formulations showed that the quadratic model best fit the data. Table 1 displays the R^2^, standard deviation, and percentage coefficient of variation (CV) values for all three replies. The three-dimensional chart shows how different factors affect the vesicle size (Y1), PDI (Y2), and entrapment efficiency (%) (Y3) (Figure 1).

Response 1 (Y1): The impact of independent factors on particle size

All 17 experimental runs’ average vesicle sizes were found to fall within the minimum and highest values of 70.11 nm and 270.87 nm, respectively.

Particle Size (Y1) = +180.79 + 54.06 A − 6.70 B − 38.27 C − 6.91 AB + 9.69 AC + 29.27 BC − 15.44 A^2^ + 3.08 B^2^ + 10.07 C^2^

According to the above polynomial equation, Lipoid S 100 was discovered to be exhibiting a positive influence on vesicle size, while ethanol and sodium cholate have a negative effect on vesicle size. It was shown that as Lipoid S 100 concentration increased, the size of transethosome vesicles increased. When Lipoid S 100 concentration was increased from 70 mg (formulation 10) to 90 mg, it was noticed that the size of the vesicles rose from 70.11 ± 0.76 nm (formulation 10) to 175.65 ± 1.21 nm (formulation 3). As seen in the above equation, it was observed that ethanol has an inverse effect on the globule size. The globule size decreases upon increasing the concentration of ethanol in the transethosome from 15 to 35%. Formulation 2, with 15% ethanol, has globule size of 270.87 ± 2.01 nm, while Formulation 5, with 35% ethanol, has globule size of 189.11 ± 1.47 nm. Since sodium cholate was shown to have an inverse effect on globule size, its effect is plainly visible in the equation above. As the sodium cholate concentration was increased from 5 mg to 15 mg, the size of the transethosome vesicles decreased. Formulation 9, with 5 mg of sodium cholate, showed vesicles measuring 261.33 ± 1.04 nm, while Formulation 16, with 15 mg of sodium cholate, showed vesicles measuring 140.21 ± 1.29 nm.

The possible reasons for the influence of each independent factor on particle size include the following. At high ethanol concentrations and above specific levels, the partial solubilization of the lipid bilayers may cause the vesicular bilayers of TEs to become permeable, resulting in a considerable fall in EE% and particle size. A further rise in its concentration would result in the dissolution of vesicles. It was discovered that low ethanol concentrations increased the size of vesicles. This may be due to the vesicular bilayers having been partly solubilized (i.e., sublysis stage). At higher ethanol concentrations, the vesicles may be completely solubilized, and the TEs may no longer exist [25]. In contrast, higher concentrations of sodium cholate lead to better emulsification and solubilization, which lead to a reduction in the size of the vesicles [26]. A thicker matrix structure was formed and stiffened the vesicles when the concentration of phospholipids (PL) was raised, which presumably explains why the vesicles expanded in size. Comparable findings were also reported by Nasr et al. [27]. The drop in vesical size with increasing surfactant concentration may be attributable to the membrane’s greater elasticity and softness, which in turn enhances its reduction capacity. The findings we obtained were in agreement with those of Zaki et al. [28].

Response 2 (Y2): The impact of independent factors on PDI

All of the formulations’ PDIs were within a range between 0.262 ± 1.12 and 0.934 ± 0.29, with the average value of 0.432 ± 0.35 (Table 2).

PDI (Y2) = +0.2770 − 0.0509 A − 0.0080 B + 0.0896 C − 0.0383 AB − 0.0450 AC − 0.0012 BC + 0.3898 A^2^ + 0.2000 B^2^ − 0.1132 C^2^

The impact of the independent variables on the PDI is shown by the equation above. It was discovered that lipoid S 100 and ethanol have a negative impact on the PDI. The PDI of HTC-TES reduced when the quantities of lipoid S 100 and ethanol were raised from 70 to 110 mg and 15 to 35%, respectively. Sodium cholate, however, had a favorable impact on PDI. When the sodium cholate was increased from 5 to 10 mg, the PDI value increased. These results are in accordance with findings of Mishra et al. [29]. The increase in PDI with the increase in sodium cholate concentration is because at high SC concentrations, vesicle bilayer surface tension and interfacial tension are reduced, which reduces the distance between vesicle bilayers. However, when the concentration of bile salt is increased, it has a tendency to agglomerate, which raises the PDI [30]. In contrast, the decrease in PDI value with increases in concentration of lipid and ethanol may be due to reduction in particle size at higher ethanol concentration and smoothness of vesicles with higher lipid content leading to less agglomeration of particles, thereby increasing the homogeneity.

Response 3 (Y3): The impact of independent variables on entrapment efficiency

All formulations’ entrapment efficiencies ranged from a minimum of 39.23 ± 0.68% to a maximum of 80.14 ± 0.53%, with an average value of 57.24%. (Table 1).

Entrapment efficiency (Y3) = +87.00 + 3.03 A − 0.8588 B + 2.75 C + 2.06 AB + 1.72 AC + 1.77 BC − 6.87 A^2^ − 7.25 B^2^ − 24.75 C^2^

The results of above equation reveal that the concentration of Lipoid S 100 and sodium cholate had a beneficial impact on the HTC’s ability to be encapsulated, whilst the other variable, ethanol, had a non-favorable impact. The inherent lipophilic nature of HTC may be the cause of the steady increase in entrapment efficiency with an increase in Lipoid S 100 concentration, since the lipophilic drug would be drawn to the lipophilic phase and be deposited there. In contrast to Formulation 15, which included 80 mg of Lipoid S 100 and displayed an entrapment efficiency of 87.11 ± 1.34%, Formulation 8, which contained 70 mg of Lipoid S 100, showed an entrapment efficiency of 65.31 ± 1.84%. Ethanol had a negative impact on how well HTC was trapped in transethosomes. Formulation 2, containing 15% ethanol, has entrapment efficiency of 55.21 ± 2.11%. This was higher than Formulation 5’s entrapment efficiency of 50.12 ± 1.24%, and Formulation 5 contained 35% ethanol. For Formulations 1 and 8, similar outcomes were attained. Formulation 1 (with 25% ethanol) had an entrapment effectiveness of 87.89 ± 1.31%, whereas Formulation 8 (with 35% ethanol) had an entrapment efficacy of 65.31 ± 1.09%. Both Formulations 4 and 7 had similar outcomes, with Formulation 4’s entrapment efficiency being 71.31 ± 1.01% and Formulation 7’s being 52.43 ± 1.14 (Table 1). This might be because the vesicles leak more when the ethanol content is higher [23]. As sodium cholate concentration was increased, entrapment effectiveness also increased (Figure 1B). While Formulation 4 (sodium cholate 10 mg) showed an entrapment efficiency of 71.31 ± 1.01%, Formulation 2 (sodium cholate 5 mg) showed a value of 55.21 ± 1.04%.

Growth in vesicle size due to incorporation of a higher quantity of phospholipid may account for the initial increase in HTC entrapment in the presence of low concentrations of ethanol. Over a certain increase in concentration of ethanol, entrapment efficiency drops because ethanol causes pores to develop in phospholipid bilayers, whereas reduced particle size occurs at low lipid concentrations. Increasing the lipid concentration improves entrapment efficiency, up to a point. As sodium deoxycholate may “solubilize” and “hold” the HTC in the lipid bilayer, its presence likely contributes to a higher encapsulation efficiency for the TEs [31].

#### Optimized Formula

The optimized formula obtained from BBD for formulating HTC-TES was lipoid (85.51 mg), ethanol (24.65%), sodium cholate (11.12 mg), and HTC (1 mg/mL). Optimized formulation was further characterized for various parameters. The chosen formulation for the optimized TES is formulation code F1, which contains lipoid (mg) (A) 90, ethanol (%) (B) 25, and sodium cholate (mg) (C) 10.

### 2.2. Size, Polydispersity, and % Entrapment of the Optimized Formulation

**Figure 2:**
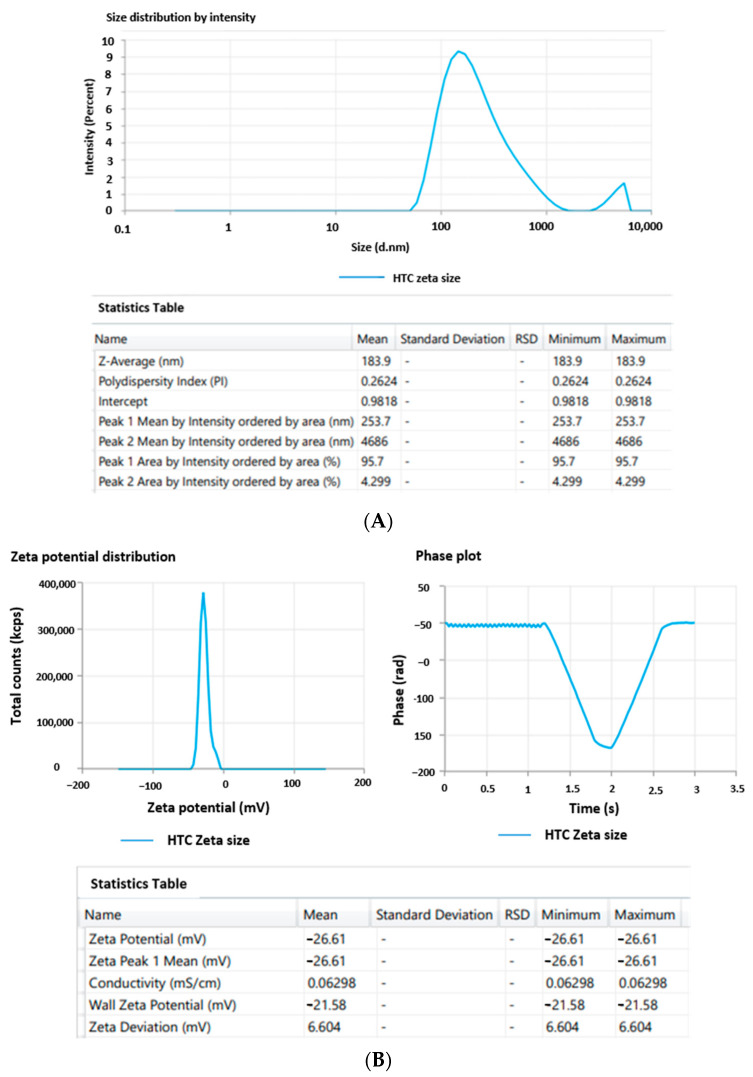
(**A**) Average particle size distribution; (**B**) average zeta potential.

According to the values provided by BBD, the optimized formulation HTC-TES was carefully developed based on the parameters of achieving the minimum particle size and PDI with maximum encapsulation efficiency. Particle size distribution (PDI) was 0.262 ± 0.05 and mean particle size was 183.9 ± 2.34 nm for the optimized TES formulation. Particles tended to homogenize as their PDI value approached zero. As a number, PDI may be anywhere from 0 (representing a completely uniform sample with regard to particle size) to 1 (representing a very uneven, highly polydisperse sample with multiple particle size populations). The fact that our PDI was so close to zero indicated that the formulation was very consistent throughout. The optimized TES formulation’s mean particle size and zeta potential curve are presented in Figure 2A,B, respectively. Stability of the formulation was confirmed by measuring the new, enhanced TES zeta potential, which came in at −26.61 ± 1.11 mV. The negative value of zeta potential is due to the use of bile salt, i.e., sodium cholate. When the zeta potential of a nanoparticle is between −10 and +10 mV, it is said to be about neutral However, when it is larger than +30 mV or lower than −30 mV, it is said to be highly cationic or anionic and considered to be highly stable, as there is repulsion between particles that prevents agglomeration [32]. The calculated entrapment efficiency Sof the improved TES system was 87.79 ± 1.84%.

### 2.3. Morphological Characteristics of Optimized TES

**Figure 3:**
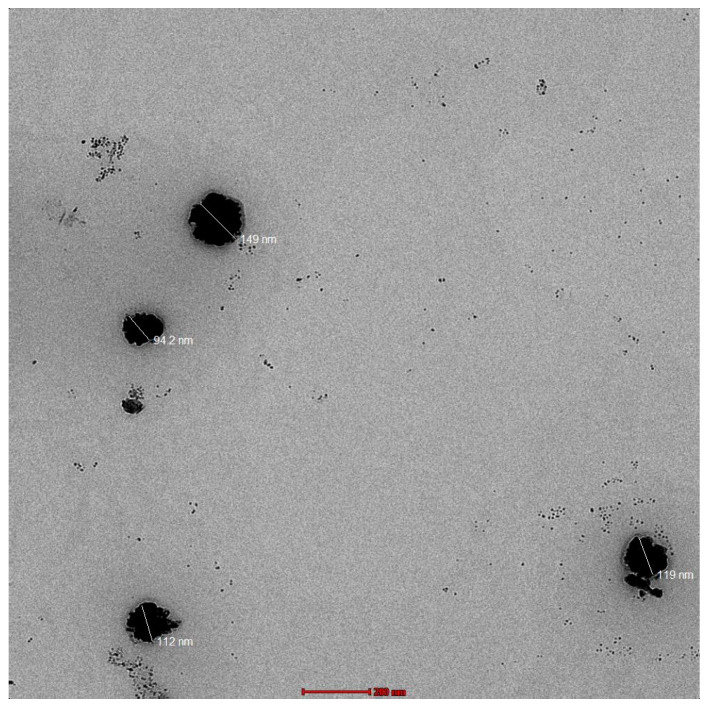
Morphology of TES.

Transmission electron microscopy (TEM) analysis of the optimized HTC-TES formulation at 28,000× demonstrated that the produced vesicles are well-identified sealed structures that are both spherical and of a constant size range (Figure 3). HTC was entirely entrapped in the vesicular structure, as evidenced by the TEM microscope image, which exhibited no crystallinity.

### 2.4. Evaluation of HTC-Based TES Gel (HTC-TESG)

The produced HTC-TESG had numerous physical properties evaluated, and the outcomes are shown in Table 2. The optimized HTC-TESG was visually appealing and consistent, since no abrasive particles were present during the process. The dermal gel that was developed has a pH of 7.28 ± 0.61, putting it in a safe range compatible with human skin. The ease with which the gel may be applied to the affected area by the patient is also an important consideration. Spreadability of the gel formulation (HTC-TESG) was 17.14 ± 3.10 g·cm/s, and extrudability was 4.48 ± 0.41 gm. The higher negative value of cohesiveness shows the stiffness of the prepared gel, whereas, the good spreadability shows better application of gel onto the surface, and good extrudability confirms the easy and uniform flow of gel from the container.

### 2.5. Texture Analysis

**Figure 4:**
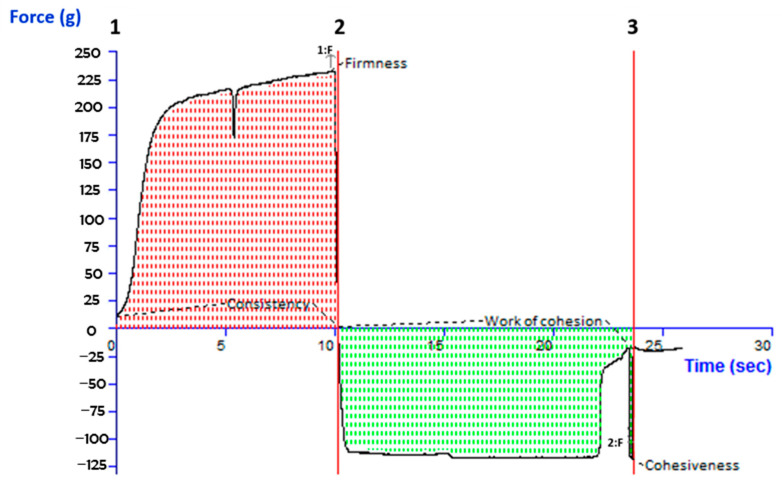
Texture analysis diagram showing firmness, consistency, cohesiveness, and work of cohesion of optimized transethosomal gel.

Texture analysis criteria including cohesion, consistency, stiffness, and viscosity index were evaluated for the HTC-TES gel formulation. According to reports, the manufactured topical gel formulation exhibited the following properties: cohesiveness (−118.29), consistency (1949.18), firmness (232.65), and work of cohesion (−1431.70). As seen in Figure 4, the software generated a report detailing its texture analysis.

### 2.6. In Vitro Drug Release

**Figure 5:**
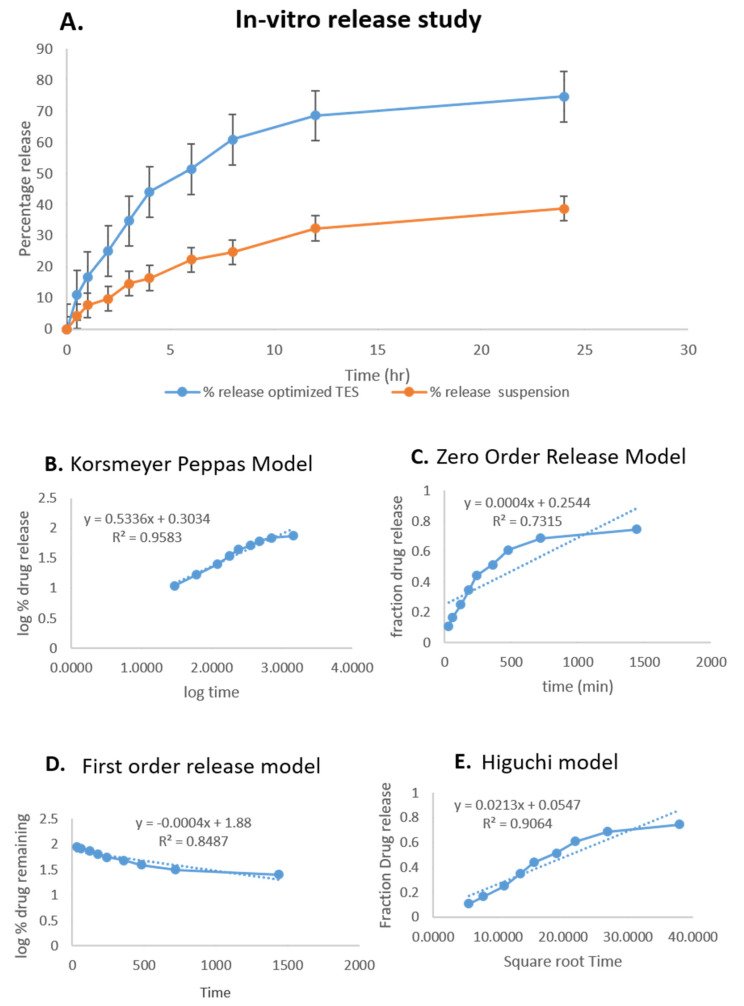
(**A**) Comparative in vitro release profiles and in vitro release kinetics graphs of HTC-TES at pH 7.4. (**B**) Korsmeyer–Peppas model. (**C**) first-order model. (**D**) zero-order model. (**E**) Higuchi model.

In a study comparing the release behavior of optimized HTC-TES and HTC conventional formulation (HTC-CF) at 37 ± 0.5 °C under continuous stirring at 100 RPM using the dialysis bag method, the percentage of HTC released by the optimized HTC-TES was substantially higher, at 74.67 ± 0.22%, than that released by the HTC-CF, at 38.75 ± 0.23% (Figure 5A). Our research has demonstrated that this TES platform may be used to safely and effectively administer HTC, with the entrapped medication being released gradually over the course of 24 h. The in vitro release data were analyzed using several different models, including the zero-order, first-order, Higuchi, and Korsmeyer–Peppas models. We selected the highest-valued correlation coefficient (R^2^) to establish the optimal release schedule. When comparing various models for optimal HTC-TES, it was found that the Higuchi model had the highest correlation coefficient (R^2^ = 0.9064) (Figure 5E), followed by the first-order model (R^2^ = 0.8487) (Figure 5C) and the zero-order model (R^2^ = 0.7315) (Figure 5D). The largest value of the correlation coefficient was discovered for the optimized HTC-TES, supporting the Higuchi model as the best-fit model. Finding R^2^ and *n* values of 0.9583 and 0.5336 (Figure 5B), respectively, in the Korsmeyer–Peppas model indicates that the release of HTC from the optimized HTC-TES follows non-Fickian diffusion.

### 2.7. Dermatokinetic Study

**Figure 6:**
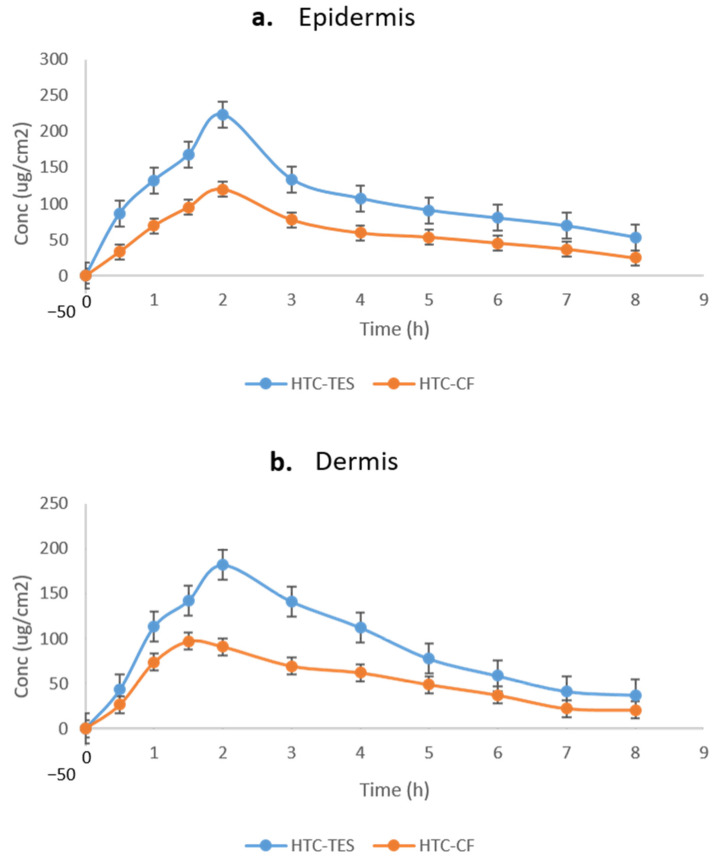
HTC concentration on (**a**) epidermis and (**b**) dermis after topical application of HTC-TES gel and HTC-CF gel on excised rat skin.

HTC distribution in the dermis and epidermis of rat skin after topical application of the developed formulation is depicted in Figure 6a,b. When comparing TES gel to HTC conventional formulation gel (HTC-CFG), it was shown that TES gel significantly boosted HTC transport in the epidermal layers (*p* < 0.05). Table 3 displays the numerical data for Cskin max, Tskin max, AUC0-8h, and constant skin disposal rate (Ke). Not only that, the epidermis and dermis also had significantly larger AUCs of HTC (*p* < 0.001 for both) [33]. The results demonstrate that the TES gel increased medication retention in both epidermal layers, which contributed to its superior efficacy in treating antimicrobial illness.

### 2.8. Confocal Laser Scanning Microscopy (CLSM)

**Figure 7:**
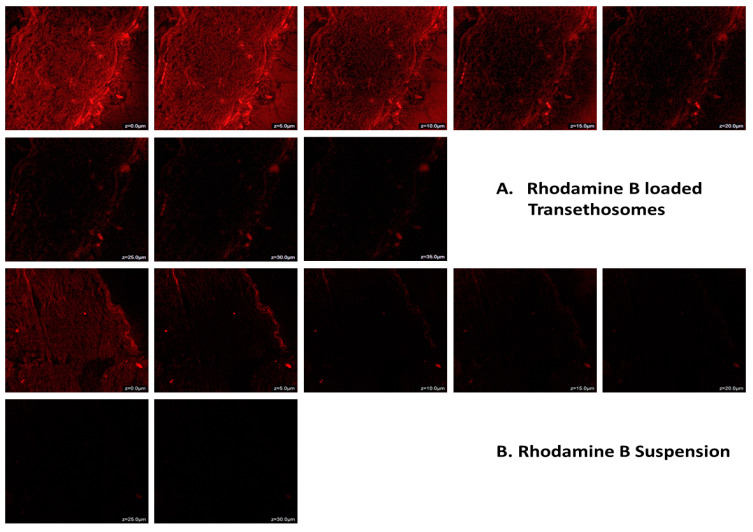
CLSM images of rat skin treated with (**A**) rhodamine B-loaded transethosomes and (**B**) rhodamine B-loaded hydroalcoholic suspension.

Confocal microscopy images showed that only the outermost layers of skin came into contact with rhodamine B hydroalcoholic solution and that the dye’s fluorescence penetrated as deeply as 0.15 μm (Figure 7B). More than 30 μm of rhodamine B-loaded TES gel was absorbed into the skin via a probe infiltration (Figure 7A). The dermal middle had the highest fluorescence intensity, indicating that the formulation was maintained at a higher concentration in the dermal lower epidermal milieu after penetrating the upper epidermis [34]. Rhodamine B was successfully transported into the dermal layers of rat skin using the newly created TES gel.

### 2.9. Antibacterial Activity of Hexatriacontane

Two different concentrations of hexatriacontane (HTC) were tested for antibacterial efficacy against two different types of bacteria: a Gram-negative strain *(E. coli*) and a Gram-positive strain (*S. aureus*). The disc diffusion method was utilized to evaluate the bacteria and determine their susceptibility. The antibacterial effects of HTC, both unloaded and loaded into the transethosome, are shown in Table 4. HTC were found to be particularly effective in inhibiting bacterial development, a major contributor to a wide variety of skin disorders. Inhibiting the development of pathogenic bacteria *(S. aureus* and *E. coli)* at a concentration of 10 mg/mL, the HTC-loaded transethosome was determined to be the most effective. Both pathogenic strains were found to be vulnerable to free HTC.

#### 2.9.1. Minimum Inhibitory Concentration of the Hexatriacontane

**Figure 8:**
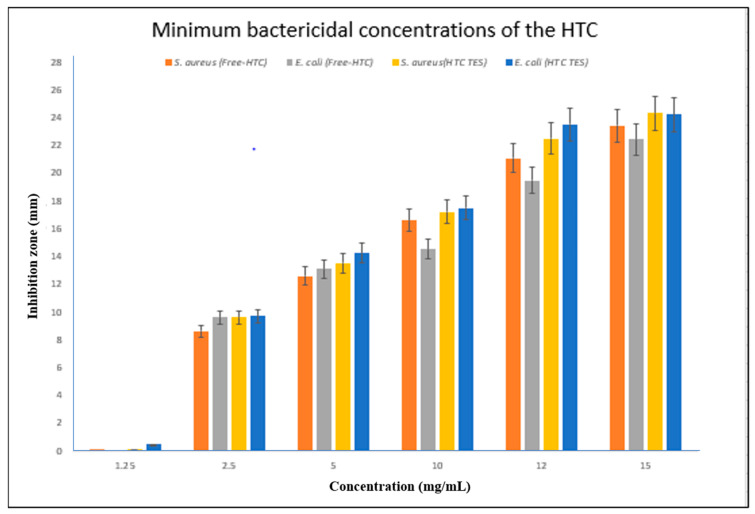
Bar graph depicting the minimal bactericidal concentrations of free HTC and HTC-TES against S. aureus and E. coli (*p*-value less than 0.05).

For the purpose of determining whether or not HTC has bacteriostatic or bactericidal capabilities, the disc diffusion method was used to determine the MIC values of both free HTC and HTC-loaded TES. Table 5 shows the effective concentrations of HTC that were tested. When tested at a concentration of 2.5 mg/mL, the inhibitory effect of free HTC was observed, with inhibition zones of 8.6 mm and 9.6 mm for *S. aureus* and *E. coli*, respectively. In contrast, the concentration of 2.5 mg/mL of HTC-loaded TES reduced the growth of bacteria, with inhibition zones of 9.6 mm and 9.71 mm for *S. aureus* and *E. coli* (Figure 8).

#### 2.9.2. Minimum Bactericidal Concentrations of HTC

The MBC is found where the inhibition zone corresponds to the MIC for the most resistant strain of bacteria. The minimum bactericidal concentration (MBC) of free HTC was determined to be 5 mg/mL against *S. aureus* and *E. coli*, while the MBC of HTC-loaded TES was also 5 mg/mL.

### 2.10. Stability

In order to estimate how long the prepared TES gel will remain stable, physiochemical stability investigations were conducted. The research showed that HTC-TES gel was preferable. According to the results of this study, HTC-TESG has no signs of phase separation and satisfies all regulatory standards for drug content, spreadability, and extrudability (Table 6). HTC-TESG formulation was found to be most stable when stored in a cool, dry place.

## 3. Conclusions

In this study, the rotary evaporator method, followed by sonication, and BBD software for optimization were used to develop HTC-TES formulations. The optimized HTC-TES formulation produced nano-sized vesicles with remarkably efficient entrapment, as was predicted. Comparing the TES gel to the rhodamine control solution, confocal laser scanning microscopy revealed that the TES gel increased rhodamine penetration across rat skin. In the dermatokinetic investigation, it was discovered that the optimized HTC-TES gel formulation had better HTC penetration than the HTC-CF formulation. The deeper skin layers are where the TES vesicular system normally produces deposits, allowing for a more steady HTC release over time and requiring fewer administrations. The antibacterial activity and MIC of HTC-loaded TES were found to be more effective as compared to the free HTC. The TES formulation was created as a drug carrier for the topical administration of HTC, as ethosomes are nanocarriers with the ability to fuse into microbial cells while increasing the effectiveness of antimicrobial drugs. Additionally, the incorporation of PEG in the gel formulation acted synergistically along with the HTC-loaded TES formulation. Based on the study’s findings, HTC-TES gel formulation showed the inhibition of the microbes (*S. aureus* and *E. coli*). Therefore, it can be concluded that HTC is a substance that is effective in the treatment of skin conditions that are caused by microbes when it is properly loaded onto the carrier system.

## 4. Materials and Methods

### 4.1. Materials

Hexatriacontane was purchased from Sigma-Aldrich (St. Louis, MO, USA), and S.D. Fine Chemicals Ltd., Mumbai, India provided the HPLC grade chloroform and methanol (Mumbai, India). Triethanolamine and polyethylene glycol (PEG) were supplied by Sigma-Aldrich (St. Louis, MO, USA). Carbopol 934 was purchased from B.S. Goodrich in Cleveland, OH, USA. An additional agent used in the experiment was of analytical quality.

### 4.2. Methods

#### 4.2.1. Development of Hexatriacontane-Loaded Transethosomes (HTC-TES)

To develop transethosomes of hexatriacontane, a rotary evaporator method was used. The known amount of Lipoid S 100, HTC, and sodium cholate was dissolved in a mixture of chloroform and methanol (2:1 *v*/*v*) and transferred to a round-bottom flask. The organic solvents were evaporated using a rotary evaporator at low pressure and vacuum after a thin coating of the lipid mixture had formed in the round-bottom flask. By removing all traces of solvents from the deposited lipid film by 4 h of vacuuming, the film was ready for use. After air drying, the deposition of lipid film was rehydrated for 1 h in a hydroethanolic solution, which was incorporated into the thin-film-containing round-bottom flask and refrigerated to produce sufficient swelling. Following that, the dispersions were ultrasonicated for 3 min in an Ultrasonicator (UP100H, Hielscher Ultrasonics GmbH, Berlin, Germany), and the resulting sonicated vesicles were extruded through a polycarbonate membrane to yield transethosomes. The vesicle size, zeta potential (charge on particles), percentage entrapment efficiency, and morphological characteristics of the vesicles that comprise the transethosomes were investigated [35].

#### 4.2.2. Preparation of HTC-Loaded Transethosomes Gel (HTC-TESG)

The developed HTC-TES system was found to be insufficiently viscous, failing to adhere to the skin of the rat. Thus, the HTC-TES system was transformed into a gel formulation (HTC-TESG). The gelling agent used was carbopol 934. In order to create the dispersion, distilled water was first gently added to the carbopol 934 (1% *w*/*w*). This mixture was then left in the dark to allow full swelling. Later, other ingredients such as polyethylene glycol 400 (PEG) (15% *w*/*w*) as a plasticizer and triethanolamine (pH modifier) were gradually added to the dispersion to further neutralize it, resulting in the formation of a transparent viscous gel. The HTC-loaded transethosomes formulation was then gradually added to the prepared gel foundation while being continuously stirred [36].

#### 4.2.3. Optimization of HTC-Loaded Transethosome Using Experimental Design

The selected independent and dependent variables for the 3-factors, 3-levels Box–Behnken design (BBD) are listed in Table 7. Different HTC-TES 17 formulations were developed and analyzed with Design Expert. According to Table 1A, vesicle size (Y1), polydispersity index (Y2), and percentage entrapment efficiency of formulation (Y3) were selected as the independent responses, whereas lipoid S 100 (A) (mg), ethanol concentration (B) (%), and sodium cholate (C) (mg) were selected as the independent factors.

### 4.3. Characterization

#### 4.3.1. Globule Size, Polydispersity Index (PDI), and Zeta Potential

Transethosomes were analyzed at 25 ±  1 °C in a Malvern zetasizer (Malvern Instruments, Worcestershire, UK) for particle size, polydispersity index, and zeta potential. Before analysis, samples were diluted using Milli-Q water, and all results were determined three times [37].

#### 4.3.2. Morphology of Transethosomes

Transmission electron microscopy was used for a morphological examination of prepared transethosomes (TEM-Tecnai, G20, Philips scientific, Amsterdam, Netherlands). On a copper grid, we dried one drop of the diluted sample. A 2% phosphotungstic acid was used to dye the dried sample. The composition was put on the copper grid and studied using transmission electron microscopy (TEM) [38].

#### 4.3.3. Entrapment Efficiency

An evaluation of the entrapment efficiency of the developed formulations was carried out using ultra-centrifugation. A total of 1 mL of the developed formulation was taken and centrifuged for 3 h at 25,000 revolutions per minute (rpm) while maintaining a 4 °C temperature (REMI cooling centrifuge, Mumbai). After completion of centrifugation, a supernatant was taken and diluted with the methanol. After that, the free HTC was examined using UV spectroscopy at 224 nm by taking blank formulation as a reference following the same procedure. We applied the following equation to determine the concentration of hexatriacontane [39].

$$\%\;Entrapment\;Efficiency=\frac{\left(total\;drug-drug\;in\;supernatant\right)}{total\;drug}\times 100$$

#### 4.3.4. In Vitro HTC Release Study

The in vitro HTC release investigation was conducted utilizing the dialysis method to examine the release of HTC from TES and suspension. HTC-TES and HTC-suspension were added (2 mg HTC) to the pre-treated and activated dialysis bag (Molecular Weight—12,000 Da), and the ends of the bag were knotted. The dialysis bags containing samples were hung in separate beakers containing 250 phosphate buffer (pH 6.4) along with 7% *v*/*v* of propylene glycol and 25% *v*/*v* methanol maintained at 37 °C. A thermostatic control was used to maintain the beaker temperature on a digital magnetic stirrer with a swirling rate of 100 rpm. At 0, 0.25, 0.5, 1, 2, 4, 12, and 24 h, a 1 mL volume of samples was taken and restored with the equal volume of a new media PBS (pH = 6.4). At 224 nm, the samples were spectrophotometrically analyzed. To determine the average results, each experiment was carried out three times. Moreover, HTC release kinetics were determined by fitting the data to various release models using their equations, given below [40,41].

$$\%\;drug\;release=\frac{conc.\left(µg/\mathrm{mL}\right)\times DF\times Volume\;of\;the\;release\;medium(mL)}{intial\;dose(µg)}\times 100$$

The equation for Korsmeyer order release is as follows:

Mt/M∞ = Kt^n^

where Mt/M∞ is a fraction of drug released at time t, k is the release rate constant, and n is the release exponent.

The equation for Higuchi order release is as follows:

Q = K_H_ t ^½^

where Q is cumulative amount of drug released at time “t”, K_H_ is Higuchi constant, and t is time in hours.

The equation for zero-order release is as follows:

Q_t_ = Q_0_ + K_0_ t

where Q_0_ is the initial amount, Q_t_ is cumulative amount of drug released at time “t”, K_0_ is zero-order release constant, and t is time in hours.

The equation for first-order release is as follows:

Log Q_t_ = Log Q_0_ + K_t_/2.303

where Q_0_ is the initial amount of drug, Q_t_ is cumulative amount of drug released at time “t”, K is the first-order release constant, and t is time in hours.

### 4.4. Evaluation of HTC-Based TES Gel

#### 4.4.1. Physical Evaluation

The color, transparency, and homogeneity of the optimized gel formulation were assessed [42].

#### 4.4.2. Texture Analysis of Gel

The software-assisted gel texture analyzer (TA.XT Plus Texture Analyzer, Stable Micro Systems Ltd., Surrey, UK) was used to examine the texture profile of developed gel. To prevent any bubble entrapment, a total of 50 mL of the produced gel was transferred to a 100 mL volume beaker and was spread uniformly. Twice, the analytical probe was placed into the beaker containing gel at a deepness of 15 mm, moving at slow speed of 2 mm per second, and allowing 20 s between each compression. The outcome was represented as a force curve that included mechanical characteristics such as cohesiveness, consistency, cohesiveness index, and hardness. The experiments were conducted in triplets [43].

#### 4.4.3. pH and HTC Content

A digital laboratory pH meter (Mettler Toledo, Model- MP 220, Greifensee, Switzerland) was used to measure the gel’s pH, and a triplicate of each experiment was performed [44]. The gel was precisely weighted (500 mg), to ascertain the HTC concentration. The weighed gel formulation was dissolved in 50 mL of phosphate-buffered saline solution (pH 6.4) in a volumetric flask. The volumetric flask was left in a shaker for two hours while being vigorously agitated to mix properly [45]. Further, the sample was taken from the dispersion, diluted, and subjected to an analysis in a UV spectrophotometer.

#### 4.4.4. Spreadability

By assessing the gel’s slipperiness and hindrance qualities, spreadability or slipperiness was evaluated. A unique arrangement was made from two clean glass slides; one of them was attached to a wooden base, whereas the other was suspended from a balance machine by a metal hook. A sample of gel weighing around 1 gm was positioned between these two glass slides. After spreading the gel, the diameter before and after the application of weight was measured (n = 3). In addition, the gel spreadability was measured using the following equation:

S = W × L/t

where S is the spreading ability (g/s), W is the given weight (g), L is the distance travelled by the glass slide, and t is the amount of time (s) required to completely divide the slides [46].

#### 4.4.5. Extrudability

The amount of gel that was forced out of a tube at a constant weight was measured in order to assess a gel’s extrusion capacity. A 20 gm gel-filled collapsible tube was used with a 1 kg fixed weight resting on its edge. When the cap was taken off, the extruded gel was collected and weighed. Extrudability was measured in gram per newton of applied force per minute [47].

#### 4.4.6. Dermatokinetics Study

The epidermis and dermis layers of rat skin were analyzed in a dermatokinetic study to assess HTC concentrations. A 1 cm^2^ piece of rat skin was cut, the hairs were removed, and the skin was mounted on a Franz diffusion cell to measure the HTC concentration in the layers of the skin. As the diffusion medium, 10 mL of PBS (pH- 6.4) was poured into the receptor. The donor compartment included the gel formulation and conventional formulation. With a magnetic stirrer, the medium in the receptor compartment was kept at 37 °C and 0.5 °C. Franz diffusion cell skin samples were collected at 0, 1, 2, 4, 6, and 8 h for this study [48]. After collecting skin samples, we washed them in 60 °C water for three minutes, then peeled off the layers, sliced them into little pieces, and stored them in methanol for 24 h to allow for the complete extraction of HTC. Multiple dermatokinetic parameters were calculated after filtering the collected HTC methanolic extract and analyzing it for HTC content using UV spectroscopy [49].

#### 4.4.7. Confocal Laser Scanning Microscopy

The permeation of hydroalcoholic rhodamine B dye solution and transethosomes containing rhodamine was monitored using confocal laser scanning microscopy. For this investigation, rat skin was prepared by washing with buffer solution and then mounted on Franz diffusion cells, with the stratum corneum facing towards the donor cell. Transethosome formulation with rhodamine added to the donor compartment was applied in vitro for six hours. A pH 7.4 phosphate buffer solution was added to the receptor compartment of the Franz diffusion cell, which was maintained at 32 ± 0.5 °C with water circulation inside the water jacket around the cell. Skin that had been gently cleaned with double-distilled water, put on a clean and dry glass slide, and treated with a drop of humectant after six hours was examined using confocal laser microscopy with excitation at 540 nm and emission at 630 nm using an argon laser beam. Skin thickness was measured with an optical z-scan using a confocal microscope [50].

### 4.5. HTC Antibacterial Activity

#### 4.5.1. Bacterial Strains

The antibacterial potency of free HTC and HTC-TES gel was examined using two different bacterial strains. One strain of Gram-positive bacteria (*S. aureus*) and one strain of Gram-negative bacteria were used in this experiment (*E. coli*). The strains were from the Prince Sattam Bin Abdulaziz University’s Biochemistry Department in Alkharj, Saudi Arabia.

#### 4.5.2. Inoculum Preparation

In Mueller–Hinton agar medium, both bacterial strains were cultured for a whole day at 35 °C. Using a spectrophotometer, bacterial growth was collected in 5 mL of sterile saline water and diluted to 107 CFU/mL at 580 nm.

#### 4.5.3. Antibacterial Activity of Free Hexatriacontane and Hexatriacontane-Loaded Transethosomes

The antibacterial activity of free hexatriacontane (F-HTC) and transethosomes loaded with hexatriacontane was determined using the disc diffusion method against both Gram-positive and Gram-negative bacteria. In order to re-dissolve the F-HTC and HTC-TES (50 mg), 2.5 mL of ethanol was used. After that, the filtrate was concentrated to a final concentration of 10 mg/disc using a Millipore filter (0.22 mm; Merck Millipore). After inoculating 10 mL of Mueller–Hinton agar media in Petri dishes with a bacterial suspension (100 mL of medium/1 mL of 107 CFU), 15 mL of seeded medium was added to bring the CFU density to 105 CFU/mL. Petri dishes were filled with Mueller–Hinton agar media, and sterile filter-paper discs containing F-HTC and HTC-TES (10 mg/mL) were placed on top of the plates. A positive control was established by using filter-paper discs saturated with 5 mg of gentamicin. After this, the plates were placed in the refrigerator for 2 h at 5 °C to allow the F-HTC and HTC-TES to diffuse before being incubated for 24 h at 35 °C. Antibacterial activity was determined by measuring the size of inhibitory zones with a Vernier caliper and recording the results [51].

#### 4.5.4. Determination of Minimum Inhibitory Concentrations of the HTC

The minimum inhibitory concentration (MIC) was defined by a low concentration of antibacterial drugs after 24 h of incubation, at which point microbial growth was inhibited. Disc diffusion was used to alter the most potent form of HTC from pure and TES forms, both of which have potent antibacterial properties at 10 mg/mL, in order to determine their MIC and analyze their efficacy in lowering food-poisoning bacterial strains. Effective HTC at several concentrations (1.25 mg/mL, 2.50 mg/mL, 5.0 mg/mL, 10.0 mg/mL, 12.0 mg/mL, 15.0 mg/mL and 5.25 mg/mL) was made by dissolving 50 mg of P-HTC and HTC-TES in 2.5 mL of ethanol, filtering it using a Millipore filter, and transferring the needed amount to sterile discs (the discs were 8 mm in diameter). Mueller–Hinton agar was used to infuse potentially harmful bacterial cultures into sterile Petri dishes. P-HTC and HTC-TES were obtained in varying concentrations and deposited onto filter-paper discs, which were then placed on top of Mueller–Hinton agar plates. After 2 h in the fridge at 5 °C, the plates were incubated at 35 °C for 24 h. The inhibition zones were measured using Vernier calipers and then compared to the concentration of the most effective HTC [51].

#### 4.5.5. Determination of Minimum Bactericidal Concentrations of the HTC

Tryptone soya agar (TSA) plates with restricted growth were cultured from two parallel streaks of plates displaying MIC inhibitory zones with low concentration. The plates were then incubated for 24 h at 35 °C. Bacteria proliferation was measured over a range of F-HTC and HTC-TES concentrations. Maximum bacterial concentration (MBC) was determined by the concentration of HTC-TES that inhibited bacterial growth on newly infected agar plates [51].

### 4.6. Stability Studies

Studies of stability were conducted to prove the assertion. The HTC-TES gel underwent stability testing under International Conference on Harmonization (ICH) Q1 A (R2) guidelines for a total of three months. The optimized HTC-TES gel was stored in a container covered with aluminum and held at a temperature of 25 ± 2 °C/60 ± 5% RH to examine its stability. Once a month throughout the period of the formulation and gel’s use, the gel’s appearance, phase separation, clarity, homogeneity, pH, spreadability, extrudability, and HTC content were measured [52,53].

## Figures and Tables

**Table 1:** (**A**) Observed responses for the HTC-loaded TES formulations during development and optimization. (**B**) Projected values produced by Design Expert software.

**(A)**						
**Formulation Code**	**A**	**B**	**C**	**Y1**	**Y2**	**Y3**
F1	90	25	10	183.95	0.262	87.89
F2	90	15	5	270.87	0.293	55.21
F3	90	25	10	175.65	0.199	86.98
F4	70	15	10	110.32	0.892	71.31
F5	90	35	15	189.11	0.298	50.12
F6	90	25	10	179.03	0.311	86.13
F7	70	25	5	170.45	0.453	52.43
F8	70	35	10	120.54	0.934	65.31
F9	110	25	5	261.33	0.432	51.98
F10	70	25	15	70.11	0.765	55.32
F11	90	35	15	175.55	0.432	58.32
F12	90	25	10	179.87	0.342	86.88
F13	110	25	15	199.76	0.564	61.76
F14	110	25	10	230.12	0.876	76.32
F15	90	25	10	185.43	0.271	87.11
F16	90	15	15	140.21	0.432	56.32
F17	110	35	10	212.71	0.765	78.54
**(B)**						
**Quadratic model**	**R^2^**	**Adjusted R^2^**		**Predicted R^2^**	**SD**	**%CV**
Response (Y1)	0.9926	0.9832		0.9041	6.57	3.66
Response (Y2)	0.9836	0.9624		0.9084	0.0482	9.61
Response (Y3)	0.9936	0.9854		0.9043	1.76	2.57

A: lipoid S 100 (mg). B: Ethanol (%). C: sodium cholate (mg). Y1: size (nm). Y2: PDI. Y3: entrapment efficiency (%). BBD: Box–Behnken experimental design. TES: Transethosome. HTC: Hexatriacontane.

**Table 2:** Physiochemical characterization of HTC-based TES gel (HTC-TESG).

**Homogeneity**	**Appearance**	**Washability**	**Phase Separation**	**Odor**
Homogeneous	Translucent	Washable	NO	NO
**Color**	**Drug content (%)**	**pH**	**Spreadability (g·cm/s)**	**Extrudability (gm)**
Off-white	89.62 ±0.35	7.28 ±0.61	17.14 ± 3.10	4.48 ± 0.41
**Cohesiveness**	**Consistency** **(gm·s)**	**Firmness (gm)**	**Work of cohesion**	
−118.29	1949.18	232.65	−1431.70	

**Table 3:** Dermatokinetic parameters (Mean ± SD) of HTC-TES-gel and HTC-CF-gel.

Dermatokinetics Parameters	HTC-TES-Gel		HTC-CF-Gel	
	Epidermis Mean ± SD	Dermis Mean ± SD	Epidermis Mean ± SD	Dermis Mean ± SD
T_skin max_ (h)	2	2	2	2
C_skin max_ (μg/cm^2^)	223.26 ± 1.26	181.9 ± 0.54	119.86 ± 1.11	96.69 ± 1.47
AUC_0–8_ (μg/cm^2^h)	869.57 ± 2.45	735.57 ± 1.22	474.58 ± 1.29	416.27 ± 0.45
Ke (h^−1^)	0.1216 ± 0.19	0.1315 ± 2.14	0.1126 ± 0.09	0.1415 ± 1.01

T_skin max_ = Time to maximum concentration, C_skin max_ = Maximum concentration, AUC = Area Under Curve, Ke = Elimination Rate Constant.

**Table 4:** Antimicrobial screening test of free hexatriacontane and HTC-TES (10 mg/mL) against some of the bacterial strains.

Hexatriacontane	Zones of Inhibition (mm)	
Bacterial strain	Gram-positive pathogenic bacteria (*S. aureus*)	Gram-negative pathogenic bacteria (*E. coli*)
Free hexatriacontane	18.46 ± 0.97	15.46 ± 0.71
Hexatriacontane-loaded transethosome	17.41 ± 0.61	14.72 ± 0.59
Gentamicin (5 μg)	19.25 ± 0.37	16.31 ± 0.68

**Table 5:** Minimum inhibitory concentrations (MICs) of HTC for *E. coli* and *S. aureus*.

Hexatriacontane Concentration [mg/mL]		Inhibition Zones (mm)	
		Gram-Positive Pathogenic Bacteria (*S. aureus*)	Gram-Negative Pathogenic Bacteria (*E. coli*)
Free hexatriacontane	1.25	0.01 ± 0.0	0.0 ± 0.0
	2.50	8.6 ± 0.51	9.6 ± 0.49
	5.0	12.6 ± 0.57	13.1 ± 0.61
	10.0	16.6 ± 0.69	14.53 ± 0.71
	12.0	21.07 ± 0.71	19.47 ± 0.76
	15.0	23.4 ± 0.51	22.41 ± 0.46
Hexatriacontane-loaded transethosomes	1.25	0.02 ± 0.0	0.46 ± 0.01
	2.50	9.6 ± 0.21	9.71 ± 0.71
	5.0	13.5 ± 0.61	14.21 ± 0.67
	10.0	17.21 ± 0.26	17.49 ± 0.71
	12.0	22.47 ± 0.51	23.51 ± 0.75
	15.0	24.32 ± 0.41	24.21 ± 0.97

**Table 6:** Stability studies for optimized HTC-TES gel stored at 25 ± 2 °C/60 ± 5%.

Evaluation Parameters for HTC-TES Gel	Months			
	Initial	1	2	3
Appearance	No change in appearance			
Phase separation	No phase separation was observed			
Homogeneity	No change in homogeneity			
pH	7.28	7.14	7.24	7.20
Drug content (%)	89.62 ± 0.35	89.57 ± 0.19	88.26 ± 0.35	87.19 ± 0.14
Spreadability	No change in spreadability			
Extrudability	No change in extrudability			

**Table 7:** List of the independent and dependent factors that were used in the BBD to develop the HTC-TES.

**Factor**			**Level Used, Actual Coded**
Independent variables	Low (−1)	Medium (0)	High (+1)
A = Lipoid S 100 (mg)	70	90	110
B = Ethanol (%)	15	25	35
C = Sodium cholate (mg)	5	10	15
**Dependent variable**			**Goal**
Y1 = Particle size (nm)			Minimize
Y2 = PDI			Minimize
Y3 = Entrapment efficiency (%)			Maximize

## Data Availability

Not applicable.
